# Self-assessed goal achievement (SAGA) after Holmium laser enucleation of the prostate (HoLEP): Association with patients' postoperative satisfaction

**DOI:** 10.1371/journal.pone.0203825

**Published:** 2018-09-13

**Authors:** Min Chul Cho, Jung Kwon Kim, Seung Beom Ha, Ja Hyeon Ku, Jae-Seung Paick

**Affiliations:** 1 Department of Urology, Seoul National University Boramae Medical Center, Seoul, Korea; 2 Department of Urology, Seoul National University Bundang Hospital, Seongnam, Korea; 3 Department of Urology, Seoul National University College of Medicine, Seoul, Korea; University Medical Center Utrecht, NETHERLANDS

## Abstract

This study aimed to determine serial changes in self-assessed goal achievement (SAGA) and treatment satisfaction after HoLEP, to identify correlations between the two, and to compare them with results assessed by traditional outcome measures. For a total of 170 patients, outcomes were evaluated serially at postoperative 1-, 3-, 6-, and 12-months using IPSS, OABSS, SAGA questionnaires and uroflowmetry. The SAGA questionnaire consisted of five questions including one open-ended question (self-assessed goals and degree of SAGA) and another question regarding treatment satisfaction. The number of self-assessed treatment goals was two or more in 74.1% of the patients. Most common treatment goal was relief from straining/hesitancy, followed by increased daytime frequency, nocturia and feeling of incomplete emptying. Degree of achievement for the first or second goal and treatment satisfaction tended to increase with time throughout the follow-up period. Patients with the greatest treatment satisfaction scores showed greater improvement by traditional outcome parameters including quality of life (QOL) index, total OABSS, maximum flow rate (Qmax), post-void residual urine volume (PVR) and bladder voiding efficiency (BVE) compared to those without treatment satisfaction. After adjusting for other influential variables, the improvements in subjective outcome parameters including total IPSS, QOL index and total OABSS were significantly associated with treatment satisfaction, but improvements in objective outcome parameters including Qmax, PVR and BVE were not. In addition, the degree of SAGA for the first goal or second goal was more predictive in determining treatment satisfaction than the traditional outcome measures. In conclusion, treatment goals of patients with lower urinary tract symptoms (LUTS)/BPH vary from individual to individual. The degree of SAGA and treatment satisfaction for HoLEP tends to increase with time throughout the follow-up period. Compared to the traditional outcome measures, the degree of goal achievement can be more predictive when assessing patient-centered outcomes such as treatment satisfaction.

## Introduction

Lower urinary tract symptoms (LUTS) are a subjective indicator of a disease or change in condition as perceived by the patient and include voiding symptoms, storage symptoms and bladder or pelvic pain [1]. Furthermore, as all patients with LUTS/benign prostatic hyperplasia (BPH) do not suffer from the same symptoms and the most bothersome symptoms differ between individuals [2,3], treatment goals and the degree of goal achievement or satisfaction with BPH treatment can vary accordingly.

Some traditional outcome measures such as the International Prostate Symptom Score (IPSS), the Overactive Bladder Symptom Score (OABSS) and uroflowmetry have been generally used to determine the efficacy of BPH treatment. However, the outcome measures could not provide important information to identify the degree of individual goal attainment or satisfaction after treatment, because they may exclude important individual concerns or focus on symptoms irrelevant to individual patients [4].

With this background, goal achievement or treatment satisfaction assessed by patients has been increasingly promoted as an important outcome measure that needs to be assessed in LUTS/BPH studies [2,3,5–7]. As the most realistic objective of BPH surgery (such as holmium laser enucleation of the prostate [HoLEP]) is to achieve treatment goals for individual patients and to meet with patients’ expectation or satisfaction, the evaluation of goal achievement or treatment satisfaction assessed by patients can be a meaningful outcome measure. However, there has been little assessment of serial change in the degree of patient’s goal achievement and treatment satisfaction after BPH surgery or correlation between the two. The objective of this study was to assess serial change in the degree of goal attainment after HoLEP. In addition, after adjusting for other influential variables, we determined a correlation between postoperative satisfaction and degree of goal achievement and compared it with a correlation between the postoperative satisfaction and results assessed by the traditional outcome measures.

## Materials and methods

This study was approved and confirmed by the Institutional Review Boards of Seoul National University Hospital, and complied with the Declaration of Helsinki (revised Edinburgh, 2000). Between September 2008 and December 2012, a total of 170 men that underwent HoLEP due to LUTS/BPH refractory to medical therapy, that did not meet any of the following exclusion criteria and for whom complete 12-month follow-up data were available, were included in this study. The exclusion criteria included previous diagnosis of neurogenic bladder, prostate or bladder carcinoma, urethral stricture disease or previous history of urinary tract surgery and a current urinary tract infection. We retrospectively reviewed our prospectively collected data. All patients provided their written informed consent.

Before surgery, all patients received baseline assessments as follows: a medical history, digital rectal examination, IPSS, OABSS, urinalysis, serum prostate-specific antigen (PSA), transrectal ultrasonography (TRUS) and urodynamic study.

HoLEP was carried out by a single surgeon (JSP), as described previously [8,9]. Briefly, we carried out enucleation that involved exposing the surgical capsule by incisions at the 5 and 7 o’clock positions just above the verumontanum, enucleating the median lobe, and then enucleating the lateral lobes. Morcellation of the enucleated lobes was carried out in a routine manner.

All patients underwent serial postoperative evaluations using the IPSS, the OABSS, uroflowmetry, post-void residual urine volume (PVR) and serum PSA at 1-, 3-, 6-, and 12-months post-surgery. Furthermore, we assessed the self-assessed goal achievement (SAGA) of each patient using an open-ended questionnaire (SAGA questionnaire) at 1-, 3-, 6-, and 12-months after HoLEP. The SAGA questionnaire was composed of five questions to assess patient’s goal attainment concerning “self-assessed goals and the degree of goal attainment” (SAGA Q1), “degree of improvement in micturition after surgery” (SAGA Q2), “degree of satisfaction with the results of HoLEP surgery” (SAGA Q3), “whether they would agree to undergo the same surgery again” (SAGA Q4), and “whether they would recommend the same surgery to others” (SAGA Q5) (Appendix 1). In Q1 of the SAGA, patients were asked to list up to five goals they had hoped to achieve through the surgery in order of priority (importance). Next, they were asked to rate how effectively the surgery helped in meeting their stated goals using a 5-point scale (1 = completely agree that the goal was achieved, 5 = completely disagree that the goal was achieved). In SAGA Q2, the degree of improvement in micturition after surgery was evaluated using a 4-point scale (1 = cured, 4 = aggravated). SAGA Q3 assessed how satisfied they were with the treatment results after HoLEP, using a 5-point scale (1 = very satisfied, 5 = very unsatisfied). At 3-, 6-, and 12-months post-HoLEP, all patients reviewed the goals that they listed in the SAGA questionnaire at the first 1-month follow-up, and completed the follow-up SAGA questionnaires. The SAGA questionnaires were collected by a study nurse who was blinded to the HoLEP surgical outcomes. Patients with a SAGA Q1 score of 1 or 2 for each goal were considered as having achieved their goal through surgery. Patients with SAGA Q3 scores of 1 or 2 were considered as being satisfied with the treatment results. BVE (%) was defined as follows: (voided volume)/(voided volume + PVR) × 100 [10].

For statistical comparisons between groups, chi square or Fisher’s exact test was used for categorical variables. Regarding continuous variables, the independent t-test and the Mann-Whitney U test were used, as indicated. For comparison between the baseline parameters and parameters obtained after surgery, the paired t-test or Wilcoxon’s signed-rank test was performed, as indicated. Spearman correlation analyses were performed to determine the association between patient’s goal attainment and treatment satisfaction. Multivariate logistical regression analyses were performed to determine the association between postoperative satisfaction and the degree of patient’s goal attainment or improvement in the traditional outcome measures (total IPSS, subtotal voiding symptoms score, subtotal storage symptoms score, quality-of-life [QOL] index, total OABSS, maximum flow rate [Qmax], PVR and BVE) with adjustment for confounding variables of age, body mass index (BMI), serum PSA, prostate volume on TRUS, bladder outlet obstruction index (BOOI) and enucleation ratio (enucleated weight/prostate volume). No one-sided test was performed. P-values < 0.05 were regarded as being statistically significant. For analyses, we used SPSS ver. 20.0 for Windows software (SPSS Inc., Chicago, IL, USA).

## Results

### Treatment goals assessed by individual patients and degree of goal achievement after HoLEP

The number of goals to achieve through the surgery was one in 44 (25.9%), two in 85 (50.0%), three in 32 (18.8%), four in 8 (4.7%) and five in 1 (0.6%). The categorization of treatment goals reported by the patients is summarized in Table 1. The most common treatment goal presented by the patients was relief of straining/hesitancy, followed by increased daytime frequency, nocturia and feeling of incomplete emptying (Table 1). Table 2 shows patient-reported outcomes throughout the follow-up period after HoLEP, according to the SAGA questionnaire. The degree of achievement for the first or second goals and the degree of treatment satisfaction at 3-, 6- and 12-months after HoLEP were significantly increased compared to those at 1 month post-operation (Table 2). Specifically, the degree of achievement for the first or second goal and satisfaction with treatment results tended to improve with time over the entire 12-month follow-up period. The greater degree of goal achievement for the first, second and the third goals was significantly associated with the greater degree of treatment satisfaction throughout the entire HoLEP follow-up period (Table 2). Furthermore, the percentage of patients who reported that they would have the same surgery again or would recommend the same surgery to others tended to increase over time throughout the HoLEP follow-up period.

**Table 1:** Summary of categorization of treatment goals listed in the SAGA questionnaire by patients.

Goals	The first goal	The second goal	The third goal	The fourth goal	The fifth goal
Number of patients (%)					
Micturition symptoms					
- Feeling of incomplete emptying	4 (2.4)	13 (10.3)	6 (14.6)	1 (11.1)	
- Increased daytime frequency	46 (27.1)	29 (23.0)	2 (4.9)		
- Intermittency		4 (3.2)	4 (9.8)		
- Urgency	8 (4.7)	9 (7.1)	5 (12.2)		
- Slow stream	10 (5.9)	9 (7.1)	2 (4.9)		
- Straining or hesitancy	66 (38.8)	35 (27.8)	14 (34.1)	6 (66.7)	
- Nocturia	30 (17.6)	21 (16.7)	7 (17.1)	2 (22.2)	1 (100.0)
- Post micturition dribble			1 (2.4)		
- Urinary incontinence		1 (0.8)			
Physical symptoms					
- Urethral pain or dysuria		3 (2.4)			
- Bladder pain	1 (0.6)	1 (0.8)			
- Hematuria	3 (1.8)				
- Hematospermia	1 (0.6)				
A desire for less medication	1 (0.6)	1 (0.8)			
**Total**	170 (100.0)	126 (100.0)	41 (100.0)	9 (100.0)	1 (100.0)

SAGA = self-assessed goal achievement

**Table 2:** Serial follow-up of degree of SAGA throughout the follow-up period after HoLEP, according to the SAGA questionnaire.

Goals	1 month	3 months	6 months	12 months
SAGA Q1				
- The first goal achievement (score)	2.16 ± 0.61 *	2.04 ± 0.56 * †	1.98 ± 0.64 * †	1.94 ± 0.57 * †
- The second goal achievement (score)	2.12 ± 0.68 *	1.99 ± 0.56 * †	1.95 ± 0.62 *†	1.98 ± 0.54 * †
- The third goal achievement (score)	1.98 ± 0.60 *	2.02 ± 0.66 *	1.88 ± 0.66 *	1.98 ± 0.60 *
- The fourth goal achievement (score)	1.67 ± 0.50	2.33 ± 1.12	2.00 ± 0.87	2.00 ± 0.50
SAGA Q2 : Outcomes for micturition symptoms				
- Cured or improved	151 (88.9)	155 (91.2)	157 (92.3)	157 (92.3)
- No change or aggravated	19 (11.1)	15 (8.9)	13 (7.7)	13 (7.7)
SAGA Q3 (score) : Satisfaction with treatment results	2.41 ± 0.68	2.15 ± 0.60 †	2.09 ± 0.57 †	2.09 ± 0.50 †
- Very satisfied	3 (1.8)	12 (7.1)	15 (8.8)	12 (7.1)
- Satisfied	110 (64.7)	127 (74.7)	129 (75.9)	132 (77.6)
- Neither satisfied nor unsatisfied	42 (24.7)	25 (14.7)	21 (12.4)	24 (14.1)
- Unsatisfied	15 (8.8)	5 (3.5)	5 (2.9)	2 (1.2)
- Very unsatisfied	0 (0.0)	1 (0.6)	0 (0.0)	0 (0.0)
SAGA Q4, No pts (%) : Same treatment again?				
- Yes	119 (70.0)	131 (77.1)	133 (78.2)	139 (81.8)
SAGA Q5, No pts (%) : Recommend to others?				
- Yes	137 (80.6)	146 (85.9)	154 (90.6)	154 (90.6)

SAGA = self-assessed goal achievement, HoLEP = holmium laser enucleation of the prostate

* p < 0.05; analyses of correlation between the SAGA Q1 scores and the SAGA Q3 scores using the Spearman correlation coefficient

† p < 0.05; comparison between the parameters obtained at 1 month after HoLEP and those at 3 or 6 or 12 months after HoLEP using the Wilcoxon’s signed-rank test

### HoLEP treatment results as assessed by traditional outcome measures

Table 3 compared the baseline characteristics and postoperative outcomes between men satisfied with their treatment and those dissatisfied with their treatment 12-months after HoLEP. Patients that were satisfied with the HoLEP treatment results had significantly larger prostate volume, lesser subtotal voiding symptom scores and greater BOOI than those unsatisfied with HoLEP. Additionally, the men with greater treatment satisfaction showed the greatest improvement in QOL index, total OABSS, Qmax, PVR and BVE compared to those dissatisfied with the treatment. The patients with greater treatment satisfaction showed the lower incidence of dysuria than those dissatisfied with the treatment. Also, when preoperative BOO was defined as BOOI of ≥ 40 on baseline urodynamic study, patients with BOO (n = 78) showed significantly greater degree of treatment satisfaction than those without BOO (n = 92) through the entire follow-up period after HoLEP. However, there was no significant difference in treatment satisfaction between patients without detrusor overactivity (n = 111) and those with detrusor overactivity (n = 59) throughout the entire follow-up period after HoLEP.

**Table 3:** Comparison of baseline characteristics and postoperative outcomes or complications between men with treatment satisfaction and those without treatment satisfaction at 12 months after HoLEP.

	Satisfied (n = 144)	Unsatisfied (n = 26)	P-value *	Total (n = 170)
Baseline characteristics				
- Age, yr	69.38 ± 6.21	67.15 ± 8.26	0.202	69.04 ± 6.59
- BMI, kg/m^2^	23.78 ± 3.11	24.26 ± 4.08	0.487	23.85 ± 3.53
- Hypertension, No. (%)	76 (52.8)	11 (42.3)	0.396	87 (51.2)
- Diabetes mellitus, No. (%)	22 (15.3)	5 (19.2)	0.570	27 (15.9)
- PSA, ng/ml	3.58 ± 4.00	3.02 ± 3.40	0.501	3.49 ± 3.91
- Total prostate vol., ml	59.49 ± 20.06	47.93 ± 14.57	**0.006**	57.72 ± 19.73
- Transition zone vol., ml	33.19 ± 18.01	22.88 ± 12.47	**0.001**	31.54 ± 17.62
- Subtotal voiding symptom score	10.96 ± 4.62	13.81 ± 4.96	**0.005**	11.41 ± 4.78
- Subtotal storage symptom score	7.14 ± 3.21	8.08 ± 4.20	0.292	7.29 ± 3.39
- Total IPSS	18.07 ± 6.67	21.81 ± 7.99	**0.012**	18.66 ± 7.00
- QOL index	3.89 ± 1.06	4.27 ± 0.79	0.085	3.95 ± 1.03
- Total OABSS	6.07 ± 3.12	4.83 ± 2.88	0.120	5.88 ± 3.10
- Qmax, ml/s	10.50 ± 3.83	11.02 ± 4.53	0.574	10.58 ± 3.94
- PVR, ml	80.95 ± 112.86	38.42 ± 35.66	**0.001**	73.73 ± 105.00
- BVE, %	73.76 ± 24.84	81.98 ± 16.22	0.055	75.09 ± 23.80
- First desire to void on UDS, ml	186.14 ± 70.38	183.04 ± 62.79	0.834	185.65 ± 69.08
- MCC on UDS, ml	373.46 ± 140.17	375.31 ± 140.26	0.951	373.75 ± 139.76
- Detrusor overactivity	52 (36.1)	7 (26.9)	0.377	59 (34.7)
- BOOI	43.18 ± 26.28	30.27 ± 19.74	**0.019**	41.12 ± 25.74
- BCI	100.10 ± 23.15	92.73 ± 19.98	0.252	98.93 ± 22.74
≥ 100	73 (50.7)	9 (34.6)		82 (48.2)
< 100	71 (49.3)	17 (65.4)		88 (51.8)
- Enucleation ratio, %	90.26 ± 42.43	80.71 ± 33.56	0.376	88.86 ± 49.91
Postoperative outcomes (change from baseline)				
- Subtotal voiding symptom score				
At 1 month after HoLEP	- 6.51 ± 4.34	- 4.91 ± 2.94	0.086	- 6.27 ± 5.28
At 3 months after HoLEP	- 8.34 ± 5.43	- 7.76 ± 5.78	0.627	- 8.25 ± 5.47
At 6 months after HoLEP	- 8.43 ± 4.69	- 5.59 ± 5.84	**0.013**	- 8.00 ± 4.96
At 12 months after HoLEP	- 6.00 ± 5.97	- 5.83 ± 5.51	0.912	- 5.98 ± 5.89
- Subtotal storage symptom score				
At 1 month after HoLEP	- 1.04 ± 4.21	- 0.31 ± 3.95	0.411	- 0.93 ± 4.17
At 3 months after HoLEP	- 2.62 ± 3.44	- 1.68 ± 5.07	0.384	- 2.46 ± 3.75
At 6 months after HoLEP	- 3.58 ± 3.47	- 1.18 ± 3.17	**0.003**	- 3.22 ± 3.53
At 12 months after HoLEP	- 3.45 ± 3.40	- 2.67 ± 4.23	0.386	- 3.34 ± 3.53
- Total IPSS				
At 1 month after HoLEP	- 7.59 ± 8.43	- 5.23 ± 7.54	0.186	- 7.21 ± 8.32
At 3 months after HoLEP	- 10.91 ± 7.56	- 9.36 ± 9.45	0.369	- 10.66 ± 7.89
At 6 months after HoLEP	- 11.97 ± 7.11	- 6.68 ± 7.29	**0.002**	- 11.16 ± 7.36
At 12 months after HoLEP	- 9.47 ± 7.87	- 8.49 ± 6.15	0.561	- 9.31 ± 7.63
- QOL index				
At 1 month after HoLEP	- 1.14 ± 1.80	- 0.42 ± 1.24	**0.016**	- 1.02 ± 1.74
At 3 months after HoLEP	- 2.06 ± 1.73	- 1.04 ± 1.51	**0.007**	- 1.90 ± 1.74
At 6 months after HoLEP	- 2.15 ± 1.65	- 0.45 ± 1.14	**< 0.001**	- 1.89 ± 1.70
At 12 months after HoLEP	- 2.18 ± 1.67	- 1.14 ± 1.53	**0.009**	- 2.02 ± 1.69
- Total OABSS				
At 1 month after HoLEP	+ 1.02 ± 4.01	+ 3.17 ± 3.35	**0.037**	+ 1.35 ± 4.03
At 3 months after HoLEP	- 1.30 ± 3.80	+ 1.76 ± 4.97	**0.004**	- 0.83 ± 4.13
At 6 months after HoLEP	- 2.65 ± 3.13	+ 1.06 ± 3.34	**< 0.001**	- 2.06 ± 3.43
At 12 months after HoLEP	- 2.60 ± 3.27	+ 0.19 ± 3.60	**0.003**	- 2.17 ± 3.46
- Qmax, ml/s				
At 1 month after HoLEP	+ 11.20 ± 8.94	+ 6.56 ± 8.97	**0.047**	+ 10.42 ± 9.07
At 3 months after HoLEP	+ 13.20 ± 9.15	+ 4.77 ± 9.18	**0.001**	+ 11.81 ± 9.63
At 6 months after HoLEP	+ 12.96 ± 8.31	+ 7.27 ± 8.86	**0.018**	+ 12.06 ± 8.60
At 12 months after HoLEP	+ 10.53 ± 8.24	+ 8.01 ± 8.56	0.289	+ 10.21 ± 8.28
- PVR, ml				
At 1 month after HoLEP *	- 67.73 ± 114.86	- 20.68 ± 30.13	**< 0.001**	- 59.78 ± 106.82
At 3 months after HoLEP *	- 77.17 ± 119.57	- 22.86 ± 38.67	**< 0.001**	- 68.66 ± 112.51
At 6 months after HoLEP *	- 74.67 ± 122.08	- 27.75 ± 41.68	**0.003**	- 66.98 ± 114.09
At 12 months after HoLEP *	- 72.33 ± 116.55	- 23.74 ± 27.58	**< 0.001**	- 65.23 ± 109.49
- BVE, (% change)				
At 1 month after HoLEP *	+ 19.81 ± 24.96	+ 7.56 ± 15.72	**0.006**	+ 17.75 ± 24.05
At 3 months after HoLEP *	+ 22.39 ± 25.65	+ 6.84 ± 11.64	**< 0.001**	+ 20.01 ± 24.65
At 6 months after HoLEP	+ 22.33 ± 28.37	+ 12.50 ± 17.86	0.071	+ 20.74 ± 27.12
At 12 months after HoLEP *	+ 21.40 ± 25.56	+ 9.70 ± 12.10	**0.006**	+ 19.87 ± 24.51
- Serum PSA, (% change)				
At 3 months after HoLEP	- 72.6%	- 57.9%	0.109	- 70.8%
At 6 months after HoLEP	- 71.8%	- 65.9%	0.901	- 71.1%
At 12 months after HoLEP	- 71.8%	- 68.2%	0.743	- 71.3%
Postoperative complications, n (%)				
- transient stress incontinence	60 (41.7%)	9 (34.6%)	0.665	69 (40.6%)
- transient urge incontinence	11 (7.6%)	2 (7.7%)	1.000	13 (7.6%)
- Urinary tract infection	2 (1.4%)	0 (0.0%)	1.000	2 (1.2%)
- Dysuria	5 (3.5%)	4 (15.4%)	**0.032**	**9 (5.3%)**
- Urethral stricture	0 (0.0%)	1 (3.8%)	0.153	1 (0.6%)
- Bladder neck contracture	2 (1.4%)	0 (0.0%)	1.000	2 (1.2%)
- Retrograde ejaculation	62 (43.1%)	6 (23.1%)	0.081	68 (40.0%)

HoLEP = holmium laser enucleation of the prostate, BMI = body mass index, PSA = prostate-specific antigen, IPSS = International Prostate Symptom Score, QOL = quality of life, OABSS = Overactive Bladder Symptom Score, Qmax = maximum urine flow rate, PVR = post-void residual urine volume, BVE = bladder voiding efficiency, UDS = urodynamic study, MCC = maximum cystometric capacity, BOOI = bladder outlet obstruction index (detrusor pressure at Qmax– 2Qmax), BCI = bladder contractility index (detrusor pressure at Qmax + 5Qmax)

* comparison between satisfied and unsatisfied groups using the independent t test or the Mann-Whitney u test, as indicate

### Correlation between postoperative satisfaction and degree of patient’s goal achievement or results assessed by traditional outcome measures

After adjusting for variables including age, BMI, serum PSA, prostate volume, BOOI and enucleation ratio, postoperative treatment satisfaction was associated with improvements in subjective outcome parameters such as total IPSS (adjusted OR: 1.086, p = 0.016), QOL index (adjusted OR: 2.179, p = 0.001) and total OABSS (adjusted OR: 1.473, p = 0.006) at each HoLEP follow-up visit, except for total IPSS 12-months postoperatively and in the total OABSS at 3-months postoperatively (Table 4). In contrast, postoperative treatment satisfaction was not associated with improvement in objective outcome parameters such as Qmax, PVR and BVE throughout the entire follow-up period, except for in Qmax at the 1-month follow-up. The degree of achievement for the first goal (adjusted OR: 12.048, p < 0.001) or the second goal (adjusted OR: 12.048, p = 0.001) was significantly associated with postoperative treatment satisfaction, after adjustment for the influential variables (Table 4).

**Table 4:** Correlation between postoperative satisfaction and patient’s goal achievement or results assessed by traditional outcome measures, after adjusted for other variables including age, body mass index, serum PSA, prostate volume on TRUS, bladder outlet obstruction index and enucleation ratio (logistic regression analysis).

	Postoperative treatment satisfaction	
Outcome parameters	Adjusted OR (95% CI)	P-value *
1 month		
- Change of total IPSS	**1.071 (1.019–1.126)**	0.007
- Change of QOL index	**1.575 (1.221–2.028)**	< 0.001
- Change of total OABSS	**1.178 (1.036–1.337)**	0.012
- Change of Qmax	**1.062 (1.000–1.127)**	0.048
- Change of PVR	1.002 (0.997–1.007)	0.436
- Change of BVE	1.020 (0.996–1.045)	0.103
- Degree of the first goal achievement	**2.985 (1.401–6.369)**	0.005
- Degree of the second goal achievement	**2.347 (1.163–4.739)**	0.017
- Degree of the third goal achievement	0.400 (0.082–1.946)	0.256
3 months		
- Change of total IPSS	**1.086 (1.017–1.159)**	0.013
- Change of QOL index	**1.508 (1.124–2.024)**	0.006
- Change of total OABSS	1.047 (0.900–1.218)	0.553
- Change of Qmax	1.065 (0.990–1.146)	0.090
- Change of PVR	1.003 (0.996–1.009)	0.469
- Change of BVE	1.000 (0.975–1.025)	0.977
- Degree of the first goal achievement	**7**.**634** (**2**.**674**–**2**1.**739**)	< 0.001
- Degree of the second goal achievement	**8**.**33**3 (1.**838**–**37**.**037**)	0.006
- Degree of the third goal achievement	0.163 (0.005–2.487)	0.163
6 months		
- Change of total IPSS	**1.160 (1.050–1.284)**	0.004
- Change of QOL index	**2.179 (1.399–3.390)**	0.001
- Change of total OABSS	**1.473 (1.122–1.934)**	0.005
- Change of Qmax	1.042 (0.944–1.151)	0.413
- Change of PVR	1.003 (0.995–1.010)	0.475
- Change of BVE	1.001 (0.971–1.032)	0.933
- Degree of the first goal achievement	**12**.**048** (**3**.**021**–**47**.**619**)	< 0.001
- Degree of the second goal achievement	**12**.**048** (**1**.**919**–**76**.**923**)	0.008
- Degree of the third goal achievement	0.591 (0.098–3.554)	0.565
12 months		
- Change of total IPSS	1.027 (0.942–1.120)	0.548
- Change of QOL index	**1.563 (1.091–2.237)**	0.015
- Change of total OABSS	**1.311 (1.056–1.629)**	0.014
- Change of Qmax	1.037 (0.950–1.132)	0.413
- Change of PVR	1.005 (0.996–1.015)	0.251
- Change of BVE	1.018 (0.981–1.056)	0.350
- Degree of the first goal achievement	**9**.**434** (**2**.**762**–**32**.**258**)	< 0.001
- Degree of the second goal achievement	**9**.**259** (**2**.1**60**–**40**.**000**)	0.003
- Degree of the third goal achievement	0.036 (0.001–1.160)	0.061

PSA = prostate-specific antigen, TRUS = transrectal ultrasonography, IPSS = International Prostate Symptom Score, QOL = quality of life, OABSS = Overactive Bladder Symptom Score, Qmax = maximum urine flow rate, PVR = post-void residual urine volume, BVE = bladder voiding efficiency

* correlation between postoperative treatment satisfaction at each follow-up visit and improvement of outcome measures at the same time point, after adjusted for other variables

## Discussion

LUTS/BPH have a major impact on health-related QOL and are associated with substantial personal and social costs [11,12]. Thus, according to the recent AUA guideline, surgery is recommended for patients with LUTS/BPH refractory to and/or unwilling to use other therapies [13]. Although the usefulness of goal achievement assessments and treatment satisfaction from individual patient perspectives has been noted by researchers [3,7,14–17], there has been a scarcity of data surrounding goal achievement and patient satisfaction after HoLEP. The results of this study address this issue with several major findings. Firstly, the treatment goals listed by the patients that underwent HoLEP varied widely from individual to individual. Secondly, the degree of achievement for the first or second goal and the degree of satisfaction with treatment results tended to improve over the duration of the entire HoLEP follow-up period. Thirdly, the greater degree of goal achievement for the first, second, and third goals was associated with a greater degree of treatment satisfaction throughout the entire HoLEP follow-up period. Fourthly, the proportion of patients reporting that they would have the same surgery again or would recommend the same surgery to others tended to increase over the postoperative follow-up period. Fifthly, after adjusting for other influential variables, the improvement in subjective outcome parameters was associated with postoperative treatment satisfaction, but the improvement in objective outcome parameters was not. Rather, given the magnitude of adjusted odds ratios in our multivariate regression analyses, the degree of achievement for the first or second goal may be more significantly associated with patient satisfaction with HoLEP than the traditional outcome measures.

A better understanding of the gap between clinicians’ assumption about patients’ need or expectation and patients’ actual need or expectation may lead to better communication with patients and improve treatment satisfaction or outcomes [15]. In this context, some previous studies have shown that the degree of goal attainment could improve treatment outcomes of medical therapies in patients with LUTS secondary to BPH or overactive bladder [3,7,14,15]. This evidence underscores the usefulness of evaluating goal achievement in helping patients to develop realistic treatment expectations and increasing treatment satisfaction. Nevertheless, to date, most studies that have evaluated treatment outcomes of medical or surgical therapies for LUTS/BPH have used traditional outcome measures rather than the tools to assess patient goal attainment [18–20]. The traditional outcome measures cannot recognize that individual patients perceive their bothersome symptoms and their specific goals or treatment responses differ. Also, the QOL question of the IPSS evaluates QOL related to the overall condition of LUTS, but not QOL specific to bothersome symptom. Under this background, we found that the degree of achievement for the first or second goal could predict the treatment outcomes or satisfaction of HoLEP more accurately than the traditional outcome measures. This may indicate the importance of the self-assessed goal attainment in treatment satisfaction after HoLEP. Considering that patients’ LUTS are heterogeneous and each individual patient’s treatment goal varies, the traditional outcome measures could not identify the most bothersome LUTS or treatment goal achievements from the individual patient’s perspective. Thus, it is not valid to assume that all patients LUTS and their postoperative treatment outcomes are similar, because even patients with the same category or severity of LUTS may have different treatment goals. Therefore, the assessment of goal achievement from the individual patient’s perspective can be useful to better manage LUTS/BPH patients and increase treatment satisfaction after HoLEP. Similarly, according to the previous study by Lee et al., only 12.8% of patients who had improvement by more than 6 points in total IPSS with medical therapies reported successful achievement, indicating that patient reported goal attainment was lower compared with the improvement in total IPSS or Qmax [3]. Also, a prospective open-label study on treatment with tolterodine ER 4 mg for 12 weeks in OAB patients showed that there was no association between the outcomes measured by patient goal achievement and those by measured from the bladder diary or patient perception of bladder control or global impression of improvement [21]. In a previous study evaluating the effectiveness of specialized geriatric interventions in the frail elderly patients, the relative efficiency and effect size of goal attainment scale was higher than those of traditional outcome measures, indicating that goal attainment scale was better at measuring clinically important change that other outcome measures [22]. Thus, the patient-centered tools to assess goal attainment may provide a better understanding of treatment outcomes or satisfaction after BPH surgeries.

For the first time, we showed that the degree of goal achievement and treatment satisfaction after HoLEP tended to increase over time throughout the entire HoLEP follow-up period. Given that the degree of improvement in the subjective and objective outcome measures, including total IPSS, QOL index, total OABSS, Qmax and BVE, tended to increase throughout the follow-up period, the continual increases in the degree of goal achievement and HoLEP treatment satisfaction with time appear to be relevant results. Consequently, the percentage of patients reporting that they would undergo the same surgery again or would recommend the surgery to others had a tendency to increase over time. Furthermore, the present study is the first report showing that a greater degree of goal achievement was associated with increased postoperative satisfaction throughout the follow-up period. Comparable to our results, a greater degree of post-treatment goal attainment for pelvic floor disorder was significantly associated with increased patient satisfaction, while reduced treatment satisfaction was strongly associated with decreased goal attainment [16,17,23]. Furthermore, Mahajan et al. demonstrated that individual patient satisfaction at 3-months after reconstructive pelvic surgery further increased at 1-year postoperatively [16]. The patients with increased satisfaction from 3-months to 1-year after surgery showed a significant correlation with increased median goal achievement over the period between assessments [16].

Interestingly, the improvement in postoperative outcome objectives such as urine flow rate, PVR and voiding efficiency were not generally associated with treatment satisfaction. As the objective parameters are clinician-centered outcomes rather than patient-centered outcomes, the gap between the patients’ and clinicians’ perceptions of treatment outcomes may contribute to the clinically insignificant associations. Furthermore, the difference could be partly explained by findings of previous studies showing that the subjective improvement of LUTS did not generally coincide with the objective improvement of urine flow rate and voiding efficiency [24,25].

The present study may be restricted by several limitations. Firstly, we did not evaluate patients’ treatment goals before HoLEP. Because the present study showed that the degree of goal attainment could be more meaningful in determining patient-centered outcomes after BPH surgeries such as patient satisfaction than traditional outcome measures, future studies need to include preoperative evaluation of patients’ treatment goals as well as postoperative assessment of goal attainment. Secondly, because this study was retrospective, it could be prone to several biases. Thirdly, a non-validated questionnaire was used to assess the degree of self-assessed goal attainment. Fourthly, because the treatment response in this study was not assessed by questions regarding QOL specific to bothersome symptom or patients’ goal, further studies including assessment of patients’ goal-specific QOL are needed. Lastly, the current study was limited by the relatively small size of the cohorts. Despite these drawbacks, we believe that the present study could provide meaningful data about the usefulness of self-assessed goal attainment to better determine patient satisfaction after HoLEP. Thus, the patient-centered tool to assess goal attainment may provide valuable information on patient-reported outcomes of BPH surgeries, in that the degree of goal achievement may better reflect treatment impact of BPH surgeries from each patient’s perspective. In clinical practice, tools for self-assessed goal attainment and assessment of QOL specific to bothersome symptom may be needed to better identify patients' specific goals or treatment responses or satisfaction with BPH surgeries from each patient’s perspective, because the tools can facilitate a personally tailored communication with clinicians regarding issues that are important to individual patients.

## Conclusion

All patients with LUTS/BPH appear to have individualized treatment goals. Our data suggest that the degree of goal achievement and treatment satisfaction tends to improve with time throughout the post-HoLEP follow-up period. Compared to traditional outcome measures, the degree of goal attainment can be more meaningful in determining patient-centered outcomes such as patient satisfaction. In particular, the objective outcome measures such as urine flow rate, PVR, and voiding efficiency are not associated with patients' treatment satisfaction. In this regard, our findings support the role of patient-centered outcome measures such as self-assessed goal attainment as an important outcome measure after BPH surgery. In addition, the assessment of patient's goal attainment is important in determining whether patients’ individual concerns are better or in understanding each patient’s perception of treatment outcomes for satisfaction after BPH surgeries. However, further long-term follow-up studies with larger cohorts are necessary to validate our findings.

## Supporting information

S1 Appendix SAGA questionnaire. (DOC)
