# Supplementary material for: Self-assessed goal achievement (SAGA) after Holmium laser enucleation of the prostate (HoLEP): Association with patients' postoperative satisfaction
Source: PLoS One. 2018 Sep 13;13(9):e0203825. doi: 10.1371/journal.pone.0203825 (PMC6136759; doi:10.1371/journal.pone.0203825)
Supplement: S1 Appendix — (DOC) [file pone.0203825.s001.doc]

Supporting Information

S1 Appendix 1_SAGA questionnaire

Self-Assessed Goal Achievement (SAGA) questionnaire

Q1. Please list up to five goals you hoped to achieve through the surgery, in order of priority (importance). Looking back at the treatment goals, which you hoped to achieve, would you rate how effectively the surgery helped you in achieving your stated goals (each goal).

■ The first goal: ________________________

1. Completely agree that the goal was achieved.

2. Somewhat agree that the goal was achieved.

3. Not sure that the goal was achieved.

4. Somewhat disagree that the goal was achieved.

5. Completely disagree that the goal was achieved.

■ The second goal: ________________________

1. Completely agree that the goal was achieved.

2. Somewhat agree that the goal was achieved.

3. Not sure that the goal was achieved.

4. Somewhat disagree that the goal was achieved.

5. Completely disagree that the goal was achieved.

■ The third goal: ________________________

1. Completely agree that the goal was achieved.

2. Somewhat agree that the goal was achieved.

3. Not sure that the goal was achieved.

4. Somewhat disagree that the goal was achieved.

5. Completely disagree that the goal was achieved.

■ The fourth goal: ________________________

1. Completely agree that the goal was achieved.

2. Somewhat agree that the goal was achieved.

3. Not sure that the goal was achieved.

4. Somewhat disagree that the goal was achieved.

5. Completely disagree that the goal was achieved.

■ The fifth goal: ________________________

1. Completely agree that the goal was achieved.

2. Somewhat agree that the goal was achieved.

3. Not sure that the goal was achieved.

4. Somewhat disagree that the goal was achieved.

5. Completely disagree that the goal was achieved.

Q2. How would you describe your micturition symptoms now compared to before surgery?

1. Cured

2. Improved

3. No change

4. Aggravated

Q3. How satisﬁed are you with the results of your treatment?”

1. Very satisfied

2. Moderately satisfied

3. Neither satisfied nor unsatisfied

4. Unsatisfied

5. Very unsatisfied

Q4. Would you agree to undergo the same surgery again?

1. Yes

2. No

Q5. Would you recommend the same surgery to others?

1. Yes

2. No
